# GTS-21, a selective alpha7 nicotinic acetylcholine receptor agonist, ameliorates diabetic nephropathy in Lepr^db/db^ mice

**DOI:** 10.1038/s41598-022-27015-y

**Published:** 2022-12-26

**Authors:** Qinghe Meng, Xinghan Tian, Junwei Li, Napat Pruekprasert, Ravi Dhawan, George G. Holz, Robert N. Cooney

**Affiliations:** 1 Department of Surgery, SUNY Upstate Medical University, 750 E Adams St., Suite 8141, Syracuse, NY 13210 USA; 2 Department of Medicine, SUNY Upstate Medical University, Syracuse, NY 13210 USA; 3 Yantai Yuhuangding Hospital, No 20 Yuhuangding East Road, Yantai, 264000 Shandong Province China

**Keywords:** Kidney diseases, Metabolic disorders, Obesity, Nephrology, Chronic kidney disease, Endocrinology, Diabetes, Type 2 diabetes

## Abstract

Diabetic nephropathy (DN) is a serious complicating factor in human type 2 diabetes mellitus (T2DM), and it commonly results in end-stage renal disease (ESRD) that requires kidney dialysis. Here, we report that the α7 nicotinic acetylcholine receptor (α7nAChR) agonist GTS-21 exerts a novel anti-inflammatory action to ameliorate DN, as studied using an inbred strain of Lepr^db/db^ mice in which hyperglycemia and obesity co-exist owing to defective leptin receptor (Lepr) signaling. For this analysis, GTS-21 was administered to 10–12 week-old male and female mice as a 4 mg/kg intraperitoneal injection, twice-a-day, for 8 weeks. Kidney function and injury owing to DN were monitored by determination of plasma levels of BUN, creatinine, KIM-1 and NGAL. Histologic analysis of glomerular hypertrophy and mesangial matrix expansion were also used to assess DN in these mice. Concurrently, renal inflammation was assessed by measuring IL-6 and HMGB1, while also quantifying renal cell apoptosis, and apoptotic signaling pathways. We found that Lepr^db/db^ mice exhibited increased markers of BUN, creatinine, NGAL, KIM-1, IL-6, cytochrome C, and HMGB-1. These abnormalities were also accompanied by histologic kidney injury (mesangial matrix expansion and apoptosis). Remarkably, all such pathologies were significantly reduced by GTS-21. Collectively, our results provide new evidence that the α7nAChR agonist GTS-21 has the ability to attenuate diabetes-induced kidney injury. Additional studies are warranted to further investigate the involvement of the vagal cholinergic anti-inflammatory reflex pathway (CAP) in ameliorating diabetic nephropathy.

## Introduction

Over 34 million people in the United States have diabetes, and the incidence of this disorder is rising world-wide (National Diabetes Statistics Report, CDC). More than 90% of these patients suffer from type 2 diabetes mellitus (T2DM), a metabolic disorder that leads to hyperglycemia accompanied by multiple severe complications including: blindness, cardiovascular disease and kidney failure^1^. In this regard, diabetic nephropathy (DN) is the leading cause of end-stage renal disease (ESRD) leading to an increased risk of mortality^2^. Although the pathogenesis of kidney disease in T2D is multifactorial, it is clear that hyperglycemia, hyperlipidemia, oxidative stress, hemodynamic disturbances, inflammation and apoptotic cell death are all major contributing factors^3,4^. Resultant kidney damage is characterized by albuminuria, podocyte loss, and histological changes that include mesangial matrix expansion and increased glomerular size^5^. Blood testing reveals that increased circulating levels of kidney injury molecule-1 (KIM-1) and neutrophil gelatinase-associated lipocalin (NGAL) are biomarkers for kidney damage in DN, and that their increased levels correlate with the severity of proteinuria in DN^6,7^. Understanding the genesis and treatment of DN is facilitated by the availability of hyperglycemic, obese, db/db mice in which there is a spontaneous point mutation in the leptin receptor (Lepr) leading to a loss of receptor signaling. These db/db mice are responsive to the α7 nicotinic acetylcholine receptor (α7nAChR) agonist GTS-21 which exerts a blood glucose-lowering effect^8^, and for this reason GTS-21 might be a previously unrecognized treatment for DN, as explored in the present study.

The db/db mouse strain develops increased fat mass by the age of four weeks, and frank hyperglycemia with albuminuria by eight weeks of age. Histologic evidence of renal injury includes glomerular hypertrophy and mesangial matrix expansion that is apparent from 8 to 16 weeks of age. Increased type IV collagen, fibronectin and laminin are also detectable in kidneys from db/db mice by four months. However, glomerular basement membrane (GBM) thickening is not seen until 12 months of age, and db/db mice do not develop mesangiolysis, nodular mesangial sclerosis or progressive renal insufficiency, as is the case in humans with DN^9^. Despite these limitations, the db/db mouse represents a good model for analysis of the early pathological changes that occur in human DN.

The present study considers the possibility that in db/db mice with DN, GTS-21 exerts a beneficial effect by emulating the vagus nerve cholinergic anti-inflammatory reflex pathway (CAP). Anatomically, the peripheral afferent vagus neurons that participate in CAP sense intraperitoneal inflammatory signals and convey this information to the brainstem^10^. Resultant reflexive efferent vagus neuron activity attenuates systemic inflammation via stimulation of α7nAChR located on cells of the immune system^10^. Importantly, the CAP pathway regulates inflammation and glycemic control in mouse models of diabetes, as well as in mouse models of experimental acute kidney injury (AKI)^10–12^. Thus, a beneficial CAP-mediated anti-inflammatory effect might be reproduced by systemically administered GTS-21 acting at the α7nAChR. With this hypothesis in mind, we examined the actions of GTS-21 on renal function and injury in the db/db model of DN. New findings presented here provide evidence that CAP and α7nAChR are relevant therapeutic targets for amelioration of DN in the context of T2D.

## Results

### Actions of GTS-21 on renal function, injury and inflammation

**Figure 1:**
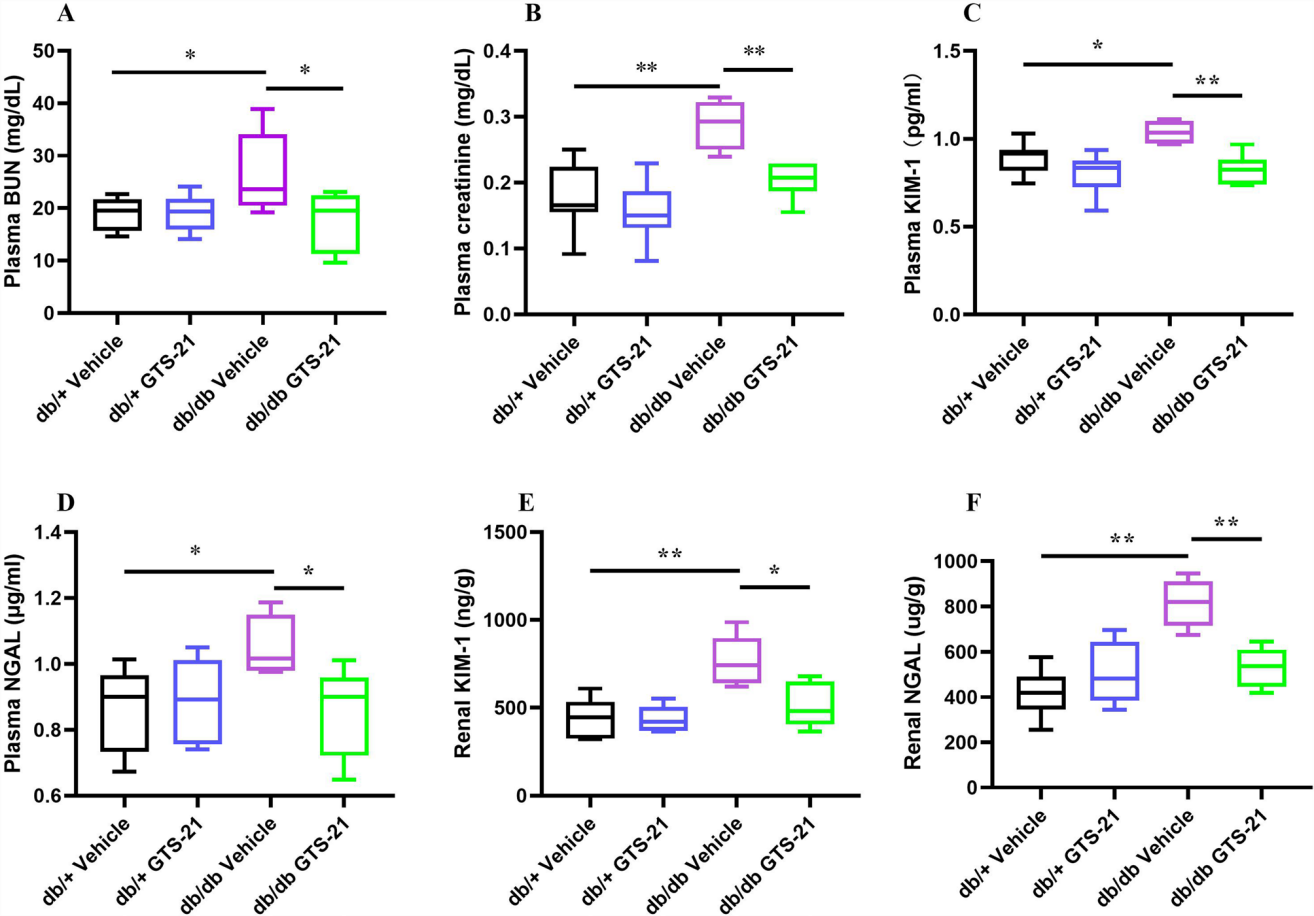
Effects of GTS-21 on renal function and injury. All mice received GTS-21 (4 mg/kg, IP, B.I.D.) or vehicle for 8 weeks. Plasma and tissue samples from kidney were collected for measuring plasma BUN (**A**) plasma creatinine (**B**), plasma KIM-1 (**C**), plasma NGAL (**D**) and renal KIM-1 (**E**) and renal NGAL (**F**) using ELISA. The data is represented as mean ± SE (n = 5–9/group), *P < 0.05, **P < 0.01.

BUN and creatinine levels in the blood are common indicators of kidney function. KIM-1 and NGAL in plasma and kidney tissue are biomarkers of acute/chronic kidney injury. In these experiments, we examine the ability of GTS-21 to attenuate the effects of T2DM on renal function, injury and inflammation. The db/db mice demonstrate a significant increase in plasma BUN (Fig. 1A) and creatinine (Fig. 1B) compared with db/+ mice (P < 0.05 vs. db/+ mice). In contrast, db/db mice treated with GTS-21 demonstrate significant reductions in plasma BUN and creatinine compared with db/db controls (P < 0.05 vs. db/db vehicle). The db/db mice also demonstrate higher levels of plasma and renal injury biomarkers KIM-1 (Fig. 1C,E) and NGAL (Fig. 1D,F) compared to db/ + vehicle controls. GTS-21 significantly attenuated the increase in plasma and renal KIM-1 and NGAL observed in the db/db mice (p < 0.05, vs. db/db vehicle).

**Figure 2:**
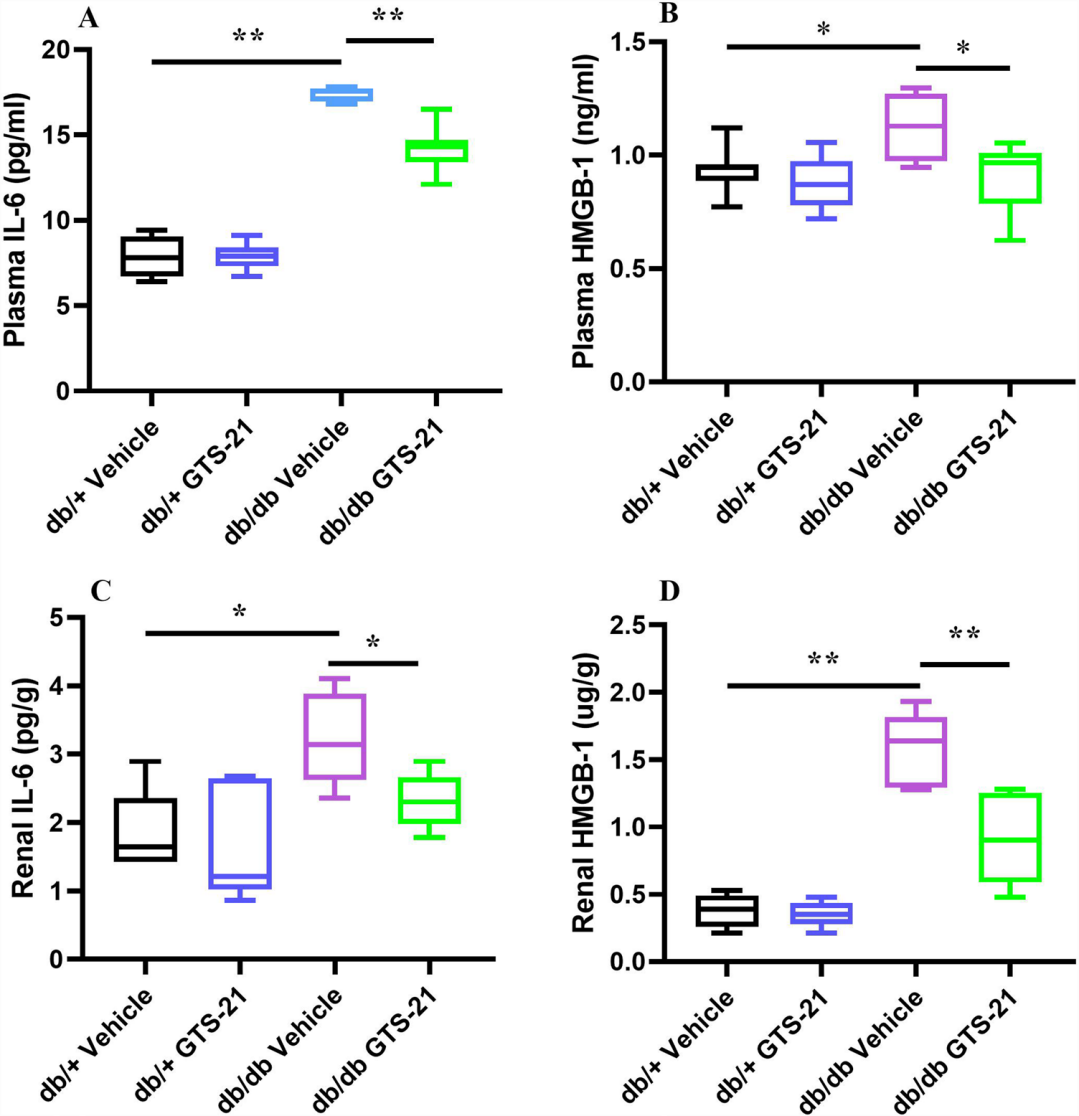
Effects of GTS-21 on inflammatory markers (IL-6 and HMGB-1). All mice received GTS-21 (4 mg/kg, IP, B.I.D.) or vehicle for 8 weeks. The plasma and protein from kidney were collected for measuring plasma IL6 (**A**), plasma HMGB-1 (**B**), renal IL-6 (**C**), and renal HMGB-1 (**D**) using ELISA. The data is represented as mean ± SE (n = 6–7/group), *P < 0.05, **P < 0.01.

IL-6 and HMGB-1 are inflammatory cytokines. Renal and plasma levels of these inflammatory markers were measured to assess the effects of GTS-21 on inflammation in the db/db mice. As shown in Fig. 2, db/db mice demonstrated increased plasma and renal IL-6 and HMGB-1 levels compared with db/+ mice. Treatment with GTS-21 significantly attenuated the elevations in IL-6 and HMGB-1 seen in the db/db mice (P < 0.05, Fig. 2). Collectively, these results provide evidence that α7nAChR activation plays an important role in improving kidney function, modulating renal injury and regulating renal inflammation in db/db mice.

### Histological assessment of diabetic nephropathy

**Figure 3:**
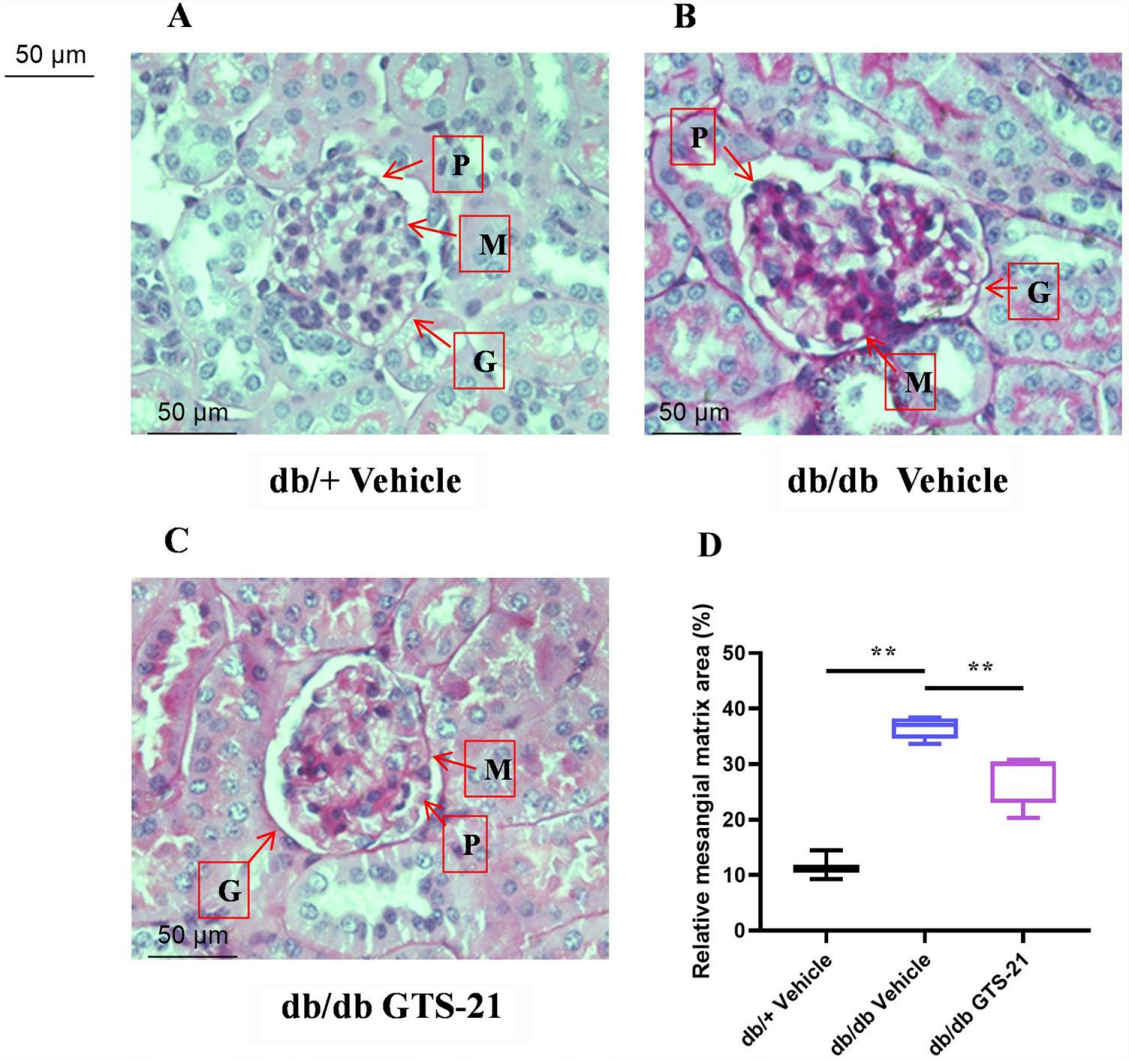
Histological assessment of diabetic nephropathy. All mice were treated with GTS-21 (4 mg/kg, IP, B.I.D.) or vehicle for 8 weeks. Kidney sections were stained with PAS to assess the glomeruli, mesangial matrix and basement membranes. Representative histological sections of the kidney are shown from db/+ mice (**A**), db/db mice (**B**) and db/db mice with GTS-21 (**C**). Magnification 400 ×. Histological changes in diabetic nephropathy were characterized by loss of podocytes (P), mesangial matrix expansion (M) and thickening of the glomerular basement membrane (G). Quantification of mesangial expansion was performed as described in Methods is shown in panel **D**. The data is represented as mean ± SE (n = 3–5/group), *P < 0.05, **P < 0.01.

Mesangial matrix expansion is characterized by an increase in extracellular matrix (ECM) consisting of type IV collagen, fibronectin and laminin in the db/db mouse model of DN. As a result of the increase in ECM, the glomerular mesangial matrix and the tubulointerstitial space are expanded. When the mesangium expands, it restricts and distorts the glomerular capillaries, resulting in reduced capillary filtration in the db/db model of DN. Mesangial matrix expansion was examined by PAS staining in the different groups as shown in Fig. 3A–C. The glomerular mesangial matrix area was quantified as described in Methods and is shown in Fig. 3D. As described by others^13^, the db/db mice demonstrate a significant increase in mesangial matrix area compared with the db/+ heterozygous controls (P < 0.05 vs. db/+ vehicle). Treatment of db/db mice with GTS-21 significantly reduced the glomerular mesangial matrix area (P < 0.05 vs. db/db vehicle). These data provide evidence that GTS-21 can attenuate the histologic changes of DN observed in the db/db mouse model.

### GTS-21 attenuates mitochondrial dysfunction and renal apoptosis

**Figure 4:**
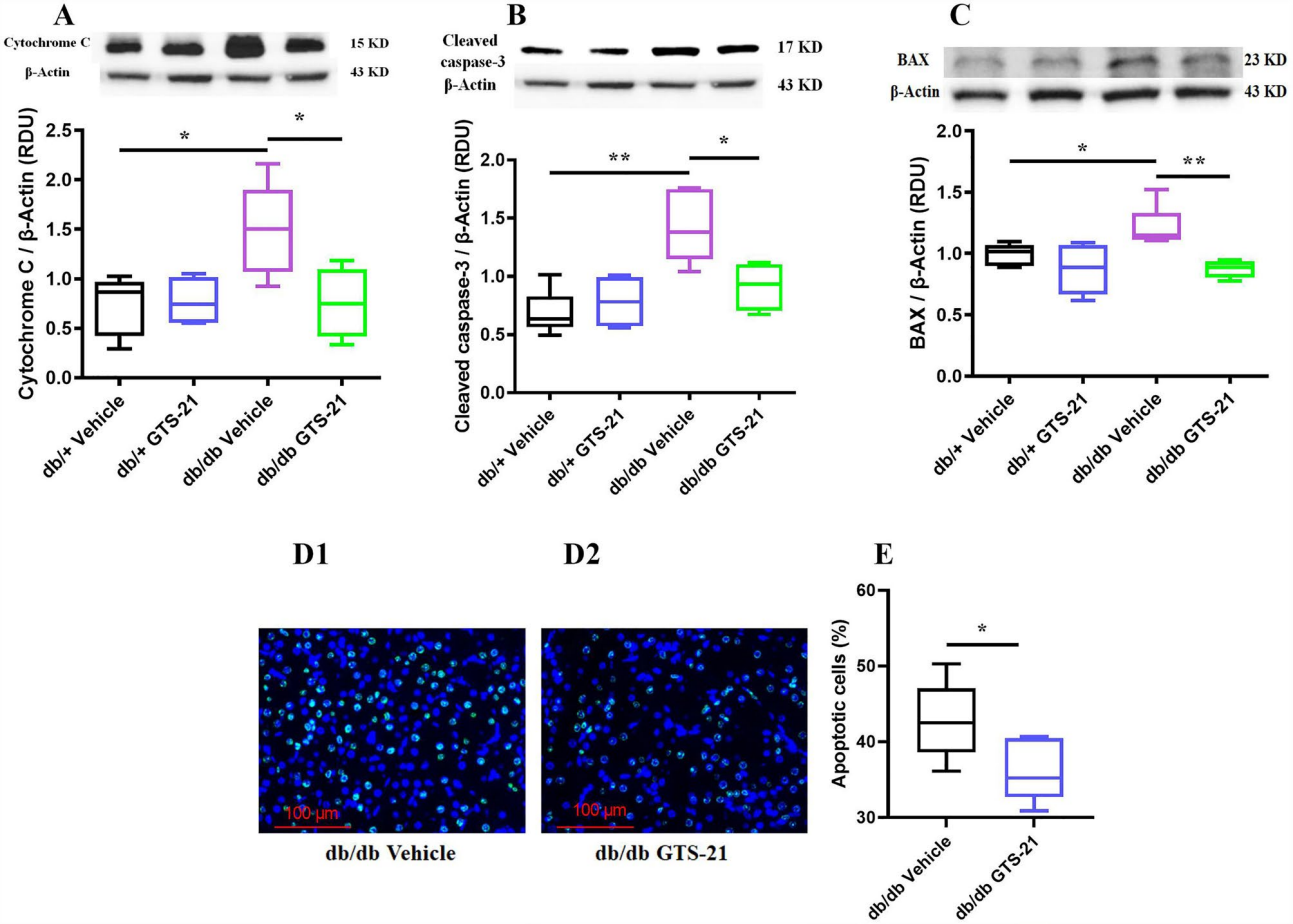
GTS-21 attenuates mitochondrial dysfunction and renal apoptosis. The db/db mice were treated with GTS-21 (4 mg/kg, IP, B.I.D.) or vehicle for 8 weeks. Western blot was used to measure apoptotic proteins (Cytochrome C in **A**, BAX in **B**, and cleaved caspase-3 in **C**). Densitometry data for the individual proteins was normalized to β-actin and is presented as relative densitometry units (RDU). Histological sections were immunofluorescence stained using TUNEL assay to detect apoptotic cells (**D1, 2**). Magnification 400 ×. Immunofluorescence stain shows TUNEL-positive cells (green), nuclei (dark blue). Quantification of TUNEL-positive cells was performed and represented in (**E**). The data is represented as mean ± SE (n = 4–6/group), *P < 0.05, **P < 0.01.

Apoptosis or programmed cell death is dysregulated in many diseases, including diabetes and contributes to organ dysfunction. In DN, damaged mitochondria release cytochrome C which initiates renal apoptosis. Cytochrome C protein levels in kidney were measured by Western Blot as shown in Fig. 4A. The increase in cytochrome C levels observed in kidneys from db/db mice was significantly attenuated by GTS-21. We examined renal apoptosis by measuring the relative abundance of pro-apoptotic proteins cleaved caspase-3 (Fig. 4B) and Bax (Fig. 4C). TUNEL staining was used to identify apoptotic cells (Fig. 4D). Renal levels of Bax (Fig. 4B) and cleaved caspase-3 (Fig. 4C) were significantly increased in kidneys from db/db mice and were attenuated by GTS-21. Consistent with this observation, the relative abundance of TUNEL-positive cells in kidneys from db/db mice were reduced by treatment with GTS-21 (Fig. 4D,E). These findings provide evidence that GTS-21 can attenuate mitochondrial damage and apoptosis in kidneys from db/db mice.

### Activation of the PI3K/AKT and p38 MAPK pathways in db/db mice

**Figure 5:**
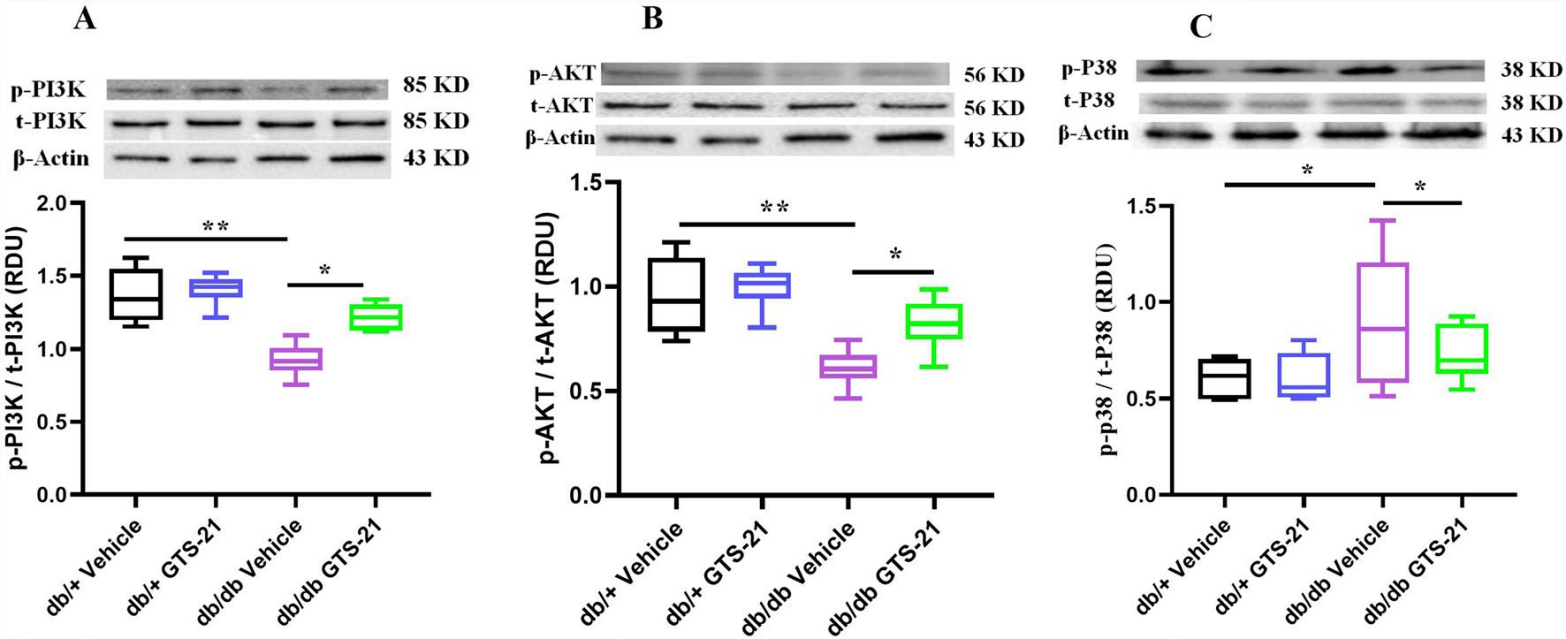
Activation of the PI3K/AKT and p38 MAPK pathways in db/db mice. Mice were treated with GTS-21 (4 mg/kg, IP, B.I.D.) or vehicle for 8 weeks. Renal lysate was prepared, then phosphorylated and total PI3K, AKT and p38 protein were assayed by Western blot. Densitometry data for phosphorylated and total PI3K (**A**), AKT (**B**) and p38 (**C**) were normalized and presented as RDU. The data is represented as mean ± SE (n = 4–5/group), *NS* no significant difference, *P < 0.05, **P < 0.01.

The PI3K/AKT pathway is an intracellular signaling pathway that regulates metabolism, proliferation, cell survival and growth in response to extracellular signals^14^. The p38 mitogen-activated protein kinase (p38 MAPK) pathway is activated by extracellular stress and regulates many cellular responses including cell differentiation, apoptosis and autophagy^15^. To investigate the role of the PI3K/AKT and p38 MAPK signaling pathways in the db/db model of DN the ratio of phosphorylated to total signaling protein in kidney was measured by Western blot analysis as an indicator of signaling pathway activation. As shown in Fig. 5A and B the ratio of phosphorylated/total PI3K and AKT is significantly decreased in kidney from db/db mice (db/db vehicle vs. db/+ vehicle, *P* < 0.05) and attenuated by treatment with GTS-21 (db/db GTS-21 vs. db/db vehicle, *P* < 0.05). In contrast, the ratio of phosphorylated/total p38 (Fig. 5C) was increased in kidney from db/db mice (db/db vehicle vs. db/+ vehicle, *P* < 0.05) and ameliorated by GTS-21 (db/db GTS-21 vs. db/db vehicle, *P* < 0.05). These results provide evidence the PI3K/AKT signaling pathway is downregulated and the p38 MAPK pathway is upregulated in kidney from db/db mice. Treatment with GTS-21 appears to attenuate these changes in PI3K/AKT and p38 MAPK signaling in kidneys from db/db mice.

## Discussion

The pathogenesis of DN is multifactorial and includes a combination of hemodynamic, metabolic and other factors. Clinically, diabetes-induced activation of the renin-angiotensin axis is managed with antihypertensive medications. Hyperglycemia and hyperlipidemia are treated with glycemic control and lipid-lowering therapies. Despite these interventions, uncontrolled inflammation, reactive oxygen species (ROS), immune cell activation, metabolic and other factors which persist can result in progressive renal injury in patients with T2DM^16,17^. The current study examines the ability of the α7nAChR agonist, GTS-21, to attenuate renal injury in the db/db mouse model.

The α7nAChR mediates many of the anti-inflammatory effects of the CAP. Studies by Tanaka et al. demonstrate vagal-mediated neuroimmune circuits which confer protection from renal injury in mice^12^. Consistent with this finding, Chatterjee et al. demonstrate the presence of the α7nAChR in mouse and human kidney cells, as well as the ability of GTS-21 to attenuate lipopolysaccharide (LPS)-induced renal inflammation and injury^18,19^. GTS-21 has also been shown to attenuate cisplatin-induced acute kidney injury^19^. Two recent reviews provide evidence for an important role of the CAP in attenuating inflammation-induced renal injury^20,21^.

We believe this to be the first study examining the effects of the CAP and α7nAChR agonist GTS-21 on DN. Our key findings include: GTS-21 improves renal function (BUN, Cr), modulates inflammation (IL-6, HMGB1), reduces kidney injury (KIM-1, NGAL), reduces histologic evidence of DN (mesangial matrix expansion) and attenuates mitochondrial damage/apoptosis (cytochrome C, Bax, cleaved caspase-3) in db/db mice with DN. These findings are associated with changes in activation of the PI3k/AKT and p38 MAPK signaling pathways in kidneys from db/db mice, and that are attenuated by GTS-21. Collectively they provide evidence α7nAChR agonists may be useful in preventing DN.

Additional studies will be necessary to fully elucidate the mechanisms by which GTS-21 attenuates DN. However, previous work from our laboratory and others provide insights into several potential mechanisms. Wang et al. showed that GTS-21 stimulates GLP-1 secretion in vitro from cultured L cells and raises circulating GLP-1 levels in vivo when administered to mice^22^. More recent studies by Meng et al. show GTS-21 improves glycemic control in db/db mice and the glycemic effects require α7nAChR and GLP-1R stimulation^8^. These results provide evidence that GTS-21-mediated improvements in glycemic control are likely to contribute to the improvements in DN in the db/db model.

Hyperglycemia contributes to the development of ROS, oxidant stress and apoptosis in renal tubular cells^23–25^. Apoptosis of renal tubular epithelial cells leads to the loss of intrinsic cells in the kidney resulting in kidney damage and decreased renal function. Several lines of evidence suggest mitochondria play an important role in the generation of ROS, are susceptible to ROS-induced damage and the release of cytochrome C from damaged mitochondria plays a role in triggering apoptosis in DN^23,26–28^. Our results showing elevations in cytochrome C, pro-apoptotic proteins Bax, cleaved caspase-3 and increased apoptosis in kidneys from db/db mice are consistent with this model. While GTS-21 attenuates mitochondrial injury and the pro-apoptotic environment, reducing apoptosis in kidneys from db/db mice, the relative importance of glycemic control vs. oxidative stress, or both will require further study.

The anti-inflammatory effects of GTS-21 and the CAP are also likely to be important mechanisms for the beneficial effects of GTS-21 on DN. The increased levels of IL-6 and HMGB1 observed in kidneys from db/db mice and their reduction by GTS-21 support an anti-inflammatory mechanism of action. The anti-inflammatory effects of α7nAChR agonists are supported by numerous studies, including those of Chatterjee et al. who showed GTS-21 attenuates inflammation-induced kidney injury by LPS^19^. Activation of the α7nAChR appears to inhibit inflammation by several mechanisms^10^. In one model, α7nAChR activation recruits and activates Janus kinase 2 (JAK2) resulting in JAK2 autophosphorylation. JAK2 recruits and phosphorylates signal transducer and activator of transcription 3 (STAT3) resulting in nuclear translocation and inhibition of the nuclear factor kappa-light-chain-enhancer of activated B cells (NF-κB) inflammatory pathway. Upregulation of interleukin-1 receptor-associated kinase M (IRAK-M) is posited as another mechanism for the anti-inflammatory effects of nicotine by Maldifassi et al.^29^. Additional studies will be necessary to determine the precise mechanisms by which GTS-21 attenuates inflammation in DN.

The PI3K pathway has been implicated in glucose regulation and previous work from our laboratory showed GTS-21 activates the PI3K/AKT/mTOR signaling pathway in L cells^22^. The reduction in phosphorylated to total PI3K and AKT suggests this signaling pathway is downregulated in kidneys from db/db mice. This finding is somewhat inconsistent with previous work suggesting a role for the PI3K/AKT pathway in interstitial fibrosis in DN^30,31^. However, the increase in PI3K/AKT signaling in GTS-21 treated kidneys from non-obese mice is consistent with our results in cultured L cells^32^. Differences in experimental conditions may help to explain conflicting results regarding the PI3K pathway in DN. In contrast, our results showing p38 MAPK activation in kidneys from db/db mice is consistent with work by Lim et al. showing that activation of the p38 MAPK pathway is important in the pathogenesis of DN in the db/db mouse model^33^. The ability of GTS-21 to attenuate the p38 MAPK pathway and DN are consistent with previous work showing inhibition of p38 MAPK activation by MAP kinase kinase (MKK)3 knockout mice also attenuates renal injury in db/db mice^33^.

There are several limitations to our study. First, mutations in the leptin receptor are a rare cause of obesity and T2DM in humans. Additionally, the db/db model is limited to modest albuminuria and mesangial expansion without overt glomerulosclerosis^5,34^. Therefore, our findings may not fully translate to the human condition. Another limitation is our failure to collect urine to assay for albuminuria, a classic finding in DN. Although we show GTS-21 ameliorates many changes in db/db kidneys with DN (e.g. inflammation, mesangial matrix expansion, apoptosis, p38 MAPK signaling) our experiments were not designed to show cause and effect or fully delineate the exact mechanisms for improvements in T2DM and DN. Additional studies will be necessary to fully elucidate the mechanisms by which α7nAChR agonists attenuate experimental DN in the db/db model.

Our findings provide evidence α7nAChR agonists attenuate diabetes-induced kidney injury in the db/db model and could serve as a novel approach to ameliorating the progression of DN in patients with T2DM.

## Methods

### Animal models

Our studies of mice were approved by the Institutional Animal Care and Use Committee of SUNY Upstate Medical University (IACUC # 423). The studies were performed in accordance with National Institutes of Health and ARRIVE guidelines for the use of laboratory animals. Heterozygous Lepr^db/+^ mice (C57BLKS genetic background) were purchased from The Jackson Laboratory (Bar Harbor, ME). Homozygous Lepr^db/db^ mice were obtained by breeding heterozygous mice so that studies could be performed in which GTS-21 action was evaluated simultaneously in Lepr^db/+^ and Lepr^db/db^ littermate mice. Breeding of mice was performed at an animal core facility located at SUNY Upstate Medical University. Mice were housed in a temperature-controlled room at 22 °C under pathogen-free conditions. Male and female mice of ages 10–12 weeks were used in this study.

### GTS-21 drug delivery protocol

Mice were divided into 4 groups at 10–12 weeks of age: (1) db/ + Vehicle, (2) db/ + GTS-21, db/db Vehicle, and (4) db/db GTS-21. These groups of mice were injected intraperitoneally (IP) twice a day (B.I.D.) for 8 weeks with the vehicle or GTS-21 (4 mg/kg) test solutions. Blood samples were collected and frozen at − 80 °C for subsequent analysis. Kidneys were collected and either flash-frozen in liquid nitrogen, or fixed in 10% neutral formalin.

### Enzyme-linked immunosorbent assays (ELISA)

Blood samples were centrifuged at 2500×*g* for 15 min, and plasma was collected for the measurements of blood urea nitrogen (ARBOR Assays), creatinine (ARBOR Assays), KIM-1 (R&D Systems), NGAL (R&D Systems), IL-6 (Thermo Fisher Scientific), and HMGB-1(MyBioSource, Inc.)

### Histology

The Periodic Acid Schiff staining of paraffin-embedded kidney sections (5 µm thick) was performed as reported previously^35^ using the kit from Thermo Scientific (Cat. #: 87007).

Briefly, (1) The section was deparaffinized and hydrated to water and then oxidized in 0.5% periodic acid solution for 5 min. (2) the section was rinsed in distilled water and placed Schiff reagent for 15 min, and then washed in lukewarm tap water for 5 min. (3) The section was with Counterstain in Mayer's hematoxylin for 1 min, and then washed in tap water for 5 min. (4) The section was dehydrated and covered using CYTOSEAL^tm^ 60 (Cat. #: 8310-4, Thermo Scientific). Digital images from 20 glomeruli per animal were used at 400 × magnification under a microscope (Nikon, Melville, NY) to assess renal pathology alternations. Relative mesangial matrix area was calculated according to the method as descripted previously using Image J^13^. Relative mesangial matrix area (%) is defined as the fractional area of mesangial matrix area (PAS-positive and nuclei-free area in the mesangium) over glomerular area (along the edge of the capillary loops of glomeruli).

### Western blot analysis

Tissues were incubated with protein lysis buffer for 30 min at 4 °C, then centrifuged at 13,000 rpm for 5 min to sediment cell debris. Supernatants were collected used for Western blot. The same amount (20 µg) of proteins were loaded and were separated by SDS-PAGE, and then transferred to PVDF membrane (Cat. #: IPVH00010, Millipore Co., Ltd. USA). The membranes were blocked with 5% nonfat milk (Cat. #: MI17200, Research Products International) in Tris-buffered TBS plus 0.5% Tween-20 (Cat. #: BP337, Fisher Scientific) for 1 h at room temperature and incubated overnight at 4 °C with the indicated primary antibodies. Total p38 (t-p38, 1:1,000, Cat. #: 8690), phospho-p38 (p-p38, 1:1,000, Cat. #: 9211) and phospho-PI3K (p-PI3K, 4228) were purchased from Cell Signaling Technologies (Danvers, MA, USA); cleaved caspase-3 (1:100, Cat. #: 56053), Bcl-2 (1:200, Cat. #: sc-7382), Bax (1:200, Cat. #: sc-7480), total AKT (t-AKT, 1:500, Cat. #: sc-81434), Phospho-AKT (p-AKT, 1:400, Cat. #: sc-377556), total PI3K (t-PI3K, 1:200, Cat. #: sc-1637) and β-actin (1:4,000, Cat. #: sc-47778) were purchased from Santa Cruz Biotechnology (Santa Cruz, CA, USA). After primary antibody incubation, the blot was incubated with HRP-conjugated secondary antibody for 1 h at room temperature. Antibody-antigen complexes were visualized with ECL (Cat. #: 34580, Thermo Scientific, IL) and analyzed quantitatively by densitometry with Image J software. The relative density of immunoreactive bands was normalized to the density of the corresponding β-actin.

### Assessment of apoptotic cells

For detecting renal apoptotic cells, deoxynucleotidyl transferase mediated dUTP nick-end labeling (TUNEL)kit (Cat. #: C10617, Invitrogen, Eugene, OR) was used according to the manufacturer’s instructions. Briefly, the sections were deparaffinized with xylene, dehydrated through a graded alcohol series to water, and treated with permeabilization solution. The labeling reaction was performed using a solution containing terminal deoxynucleotidyl transferase. After staining, the sections were mounted by fluoroshield mounting medium with DAPI (Cat. #: ab104139, Abcam Inc, Cambridge, MA) to visualize nuclei. Apoptotic cells were quantified by counting TUNEL positive cells from five randomly selected consecutive fields at 400 magnification by two experienced investigators in a blinded manner. Apoptotic index was calculated as the number of TUNEL positive cells expressed as percentage of total cells.

### Statistical analysis

The data are expressed as the mean ± SEM (with Gaussian distribution) or median ± interquartile range (with no Gaussian distribution) analyzed using GraphPad Prism 9. The Mann–Whitney test or *t* test were used to compare differences between two independent groups. One-way analysis of variance (one-way ANOVA) with Bonferroni’s multiple comparisons test or Kruskal–Wallis test with Dunn’s multiple comparison test were used to determine multiple group differences. *P* values of < 0.05 were considered significant. Data was obtained from three or more independent experiments.

## Supplementary Information

Supplementary Information.

## Data Availability

Datasets used in this study are available from the corresponding authors upon reasonable request.

## Abbreviations

AKI: Acute kidney injury
α7nAChR: α7 Nicotinic acetylcholine receptor
BUN: Blood urea nitrogen
B.I.D.: Twice a day
Cr: Creatinine
CAP: Cholinergic anti-inflammatory reflex pathway
DN: Diabetic nephropathy
ESRD: End-stage renal disease
ECM: Extracellular matrix
GBM: Glomerular basement membrane
HMGB-1: High mobility group box 1
IL-6: Interleukin 6
IP: Intraperitoneal injection
KIM-1: Kidney injury molecule-1
LPS: Lipopolysaccharide
Lepr: Leptin receptor
NGAL: Neutrophil gelatinase-associated lipocalin
p38 MAPK: p38 mitogen-activated protein kinase
ROS: Reactive oxygen species
T2DM: Type 2 diabetes

## References

[1] Hackler E (2019). Racial differences in cardiovascular biomarkers in the general population. J. Am. Heart Assoc..

[2] Wang G (2019). The analysis of risk factors for diabetic nephropathy progression and the construction of a prognostic database for chronic kidney diseases. J. Transl. Med..

[3] Alicic RZ, Rooney MT, Tuttle KR (2017). Diabetic kidney disease: Challenges, progress, and possibilities. Clin. J. Am. Soc. Nephrol..

[4] Turkmen K (2017). Inflammation, oxidative stress, apoptosis, and autophagy in diabetes mellitus and diabetic kidney disease: The Four Horsemen of the Apocalypse. Int. Urol. Nephrol..

[5] Breyer MD (2005). Mouse models of diabetic nephropathy. J. Am. Soc. Nephrol..

[6] Wasilewska A, Taranta-Janusz K, Debek W, Zoch-Zwierz W, Kuroczycka-Saniutycz E (2011). KIM-1 and NGAL: New markers of obstructive nephropathy. Pediatr. Nephrol..

[7] Edelstein CL (2008). Biomarkers of acute kidney injury. Adv. Chronic Kidney Dis..

[8] Meng Q (2022). The alpha-7 nicotinic acetylcholine receptor agonist GTS-21 engages the glucagon-like peptide-1 incretin hormone axis to lower levels of blood glucose in db/db mice. Diabetes Obes. Metab..

[9] Alpers CE, Hudkins KL (2011). Mouse models of diabetic nephropathy. Curr. Opin. Nephrol. Hypertens..

[10] Xie H (2020). Therapeutic potential of alpha7 nicotinic acetylcholine receptor agonists to combat obesity, diabetes, and inflammation. Rev. Endocr. Metab. Disord..

[11] Atkinson SJ (2016). A wandering path toward prevention for acute kidney injury. J. Clin. Invest..

[12] Tanaka S (2021). Vagus nerve stimulation activates two distinct neuroimmune circuits converging in the spleen to protect mice from kidney injury. Proc. Natl. Acad. Sci. USA.

[13] Lin Y, Sun Z (2011). Thyroid hormone ameliorates diabetic nephropathy in a mouse model of type II diabetes. J. Endocrinol..

[14] Nicholson KM, Anderson NG (2002). The protein kinase B/Akt signalling pathway in human malignancy. Cell Signal.

[15] Segales J, Perdiguero E, Munoz-Canoves P (2016). Regulation of muscle stem cell functions: A Focus on the p38 MAPK signaling pathway. Fron.t Cell Dev. Biol..

[16] Oguntibeju OO (2019). Type 2 diabetes mellitus, oxidative stress and inflammation: examining the links. Int. J. Physiol. Pathophysiol. Pharmacol..

[17] Giacco F, Brownlee M (2010). Oxidative stress and diabetic complications. Circ. Res..

[18] Chatterjee PK (2012). Nicotinic acetylcholine receptor agonists attenuate septic acute kidney injury in mice by suppressing inflammation and proteasome activity. PLoS ONE.

[19] Chatterjee PK (2017). Activation of the cholinergic anti-inflammatory pathway by GTS-21 attenuates cisplatin-induced acute kidney injury in mice. PLoS ONE.

[20] Hilderman M, Bruchfeld A (2020). The cholinergic anti-inflammatory pathway in chronic kidney disease-review and vagus nerve stimulation clinical pilot study. Nephrol. Dial. Transplant..

[21] Jarczyk J, Yard BA, Hoeger S (2019). The cholinergic anti-inflammatory pathway as a conceptual framework to treat inflammation-mediated renal injury. Kidney Blood Press Res..

[22] Wang D (2018). alpha7 nicotinic acetylcholine receptor regulates the function and viability of L cells. Endocrinology.

[23] Wagener FA, Dekker D, Berden JH, Scharstuhl A, van der Vlag J (2009). The role of reactive oxygen species in apoptosis of the diabetic kidney. Apoptosis.

[24] Wang J (2020). Acute hyperglycemia may induce renal tubular injury through mitophagy inhibition. Front. Endocrinol..

[25] Habib SL (2013). Diabetes and renal tubular cell apoptosis. World J. Diabetes.

[26] Sifuentes-Franco S, Padilla-Tejeda DE, Carrillo-Ibarra S, Miranda-Diaz AG (2018). Oxidative stress, apoptosis, and mitochondrial function in diabetic nephropathy. Int. J. Endocrinol..

[27] Lee J (2022). Natural COA water inhibits mitochondrial ROS-mediated apoptosis through Plk3 downregulation under STZ diabetic stress in pancreatic beta-cell lines. Biochem. Biophys. Rep..

[28] Pal PB, Sinha K, Sil PC (2014). Mangiferin attenuates diabetic nephropathy by inhibiting oxidative stress mediated signaling cascade, TNFalpha related and mitochondrial dependent apoptotic pathways in streptozotocin-induced diabetic rats. PLoS ONE.

[29] Maldifassi MC (2014). A new IRAK-M-mediated mechanism implicated in the anti-inflammatory effect of nicotine via alpha7 nicotinic receptors in human macrophages. PLoS ONE.

[30] Zhang Y (2021). Signaling pathways involved in diabetic renal fibrosis. Front. Cell Dev. Biol..

[31] Lu Q (2019). ROS induces epithelial-mesenchymal transition via the TGF-beta1/PI3K/Akt/mTOR pathway in diabetic nephropathy. Exp. Ther. Med..

[32] Kim H (2018). The proximal tubular alpha7 nicotinic acetylcholine receptor attenuates ischemic acute kidney injury through Akt/PKC signaling-mediated HO-1 induction. Exp. Mol. Med..

[33] Lim AK (2009). Role of MKK3-p38 MAPK signalling in the development of type 2 diabetes and renal injury in obese db/db mice. Diabetologia.

[34] Azushima K, Gurley SB, Coffman TM (2018). Modelling diabetic nephropathy in mice. Nat. Rev. Nephrol..

[35] Fu DA, Campbell-Thompson M (2017). Periodic acid-Schiff staining with diastase. Methods Mol. Biol..

