# Supplementary material for: GTS-21, a selective alpha7 nicotinic acetylcholine receptor agonist, ameliorates diabetic nephropathy in Leprdb/db mice
Source: Sci Rep. 2022 Dec 26;12:22360. doi: 10.1038/s41598-022-27015-y (PMC9792461; doi:10.1038/s41598-022-27015-y)
Supplement: Supplementary file 1 — Supplementary Information. [file 41598_2022_27015_MOESM1_ESM.pptx]

## Slide 1
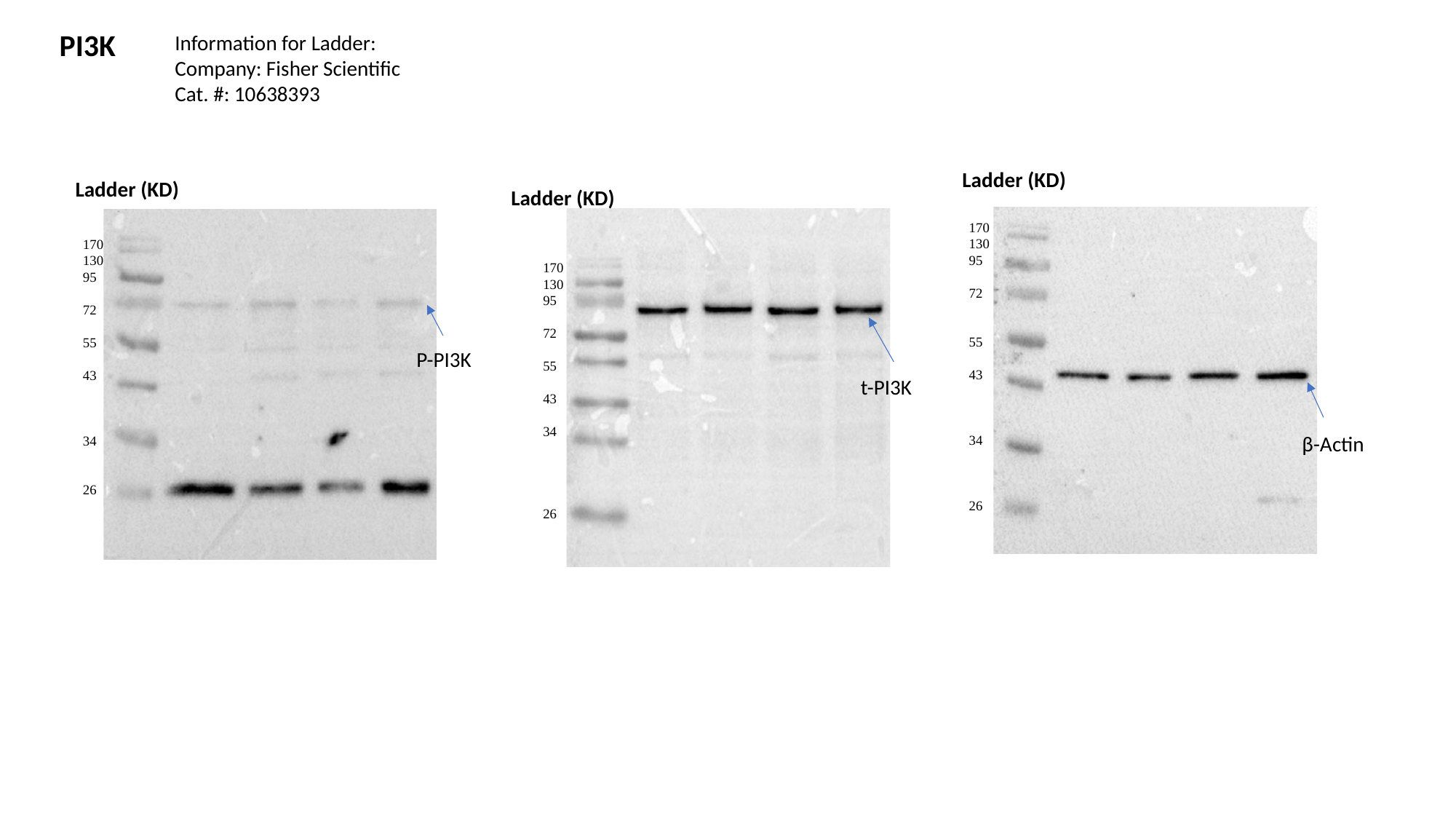

PI3K
Information for Ladder:
Company: Fisher Scientific
Cat. #: 10638393
Ladder (KD)
Ladder (KD)
Ladder (KD)
170
130
95
72
55
43
34
26
170
130
95
72
55
43
34
26
170
130
95
72
55
43
34
26
P-PI3K
t-PI3K
β-Actin

## Slide 2
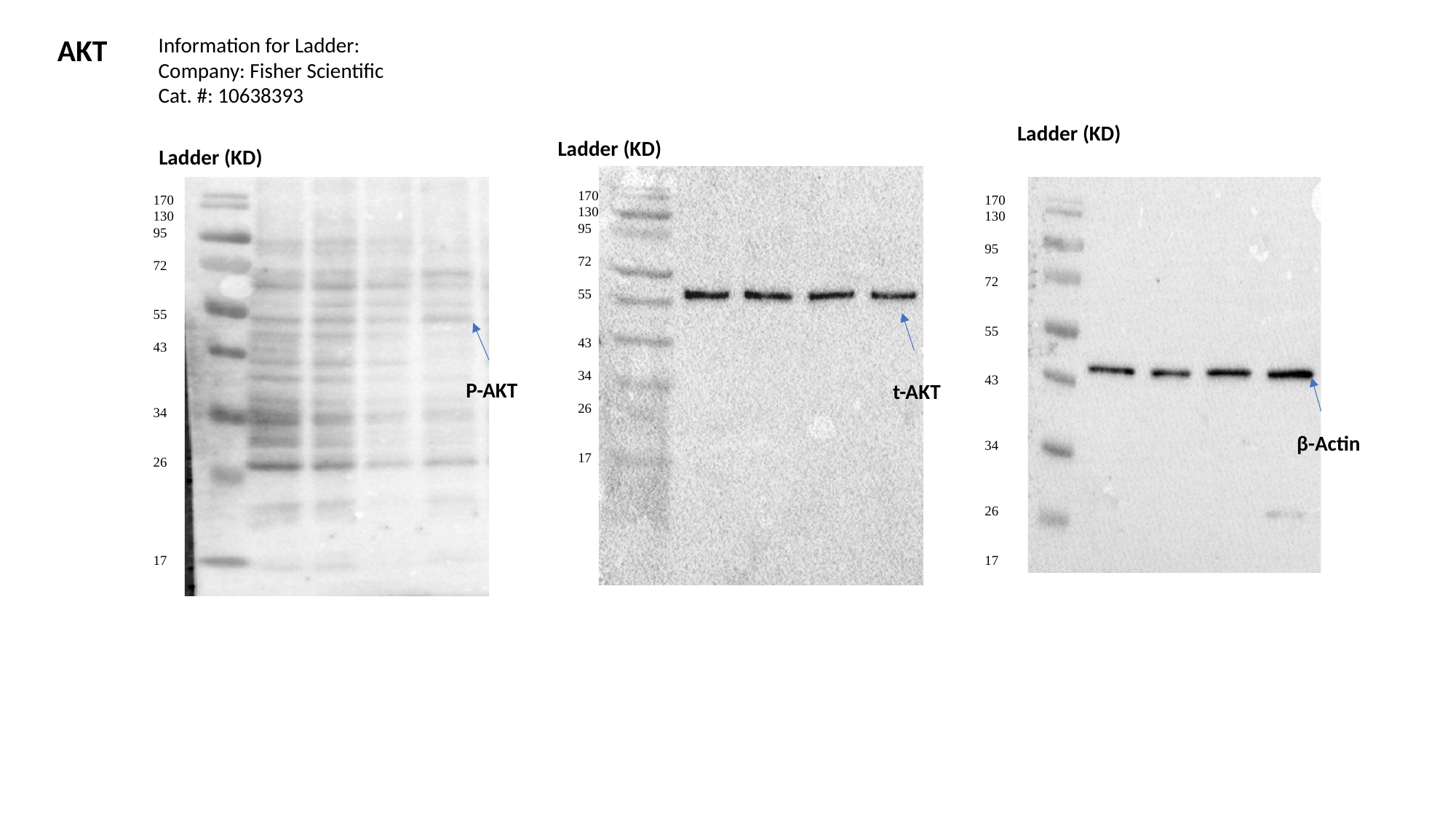

AKT
Information for Ladder:
Company: Fisher Scientific
Cat. #: 10638393
Ladder (KD)
Ladder (KD)
Ladder (KD)
170
130
95
72
55
43
34
26
17
170
130
95
72
55
43
34
26
17
170
130
95
72
55
43
34
26
17
P-AKT
t-AKT
β-Actin

## Slide 3
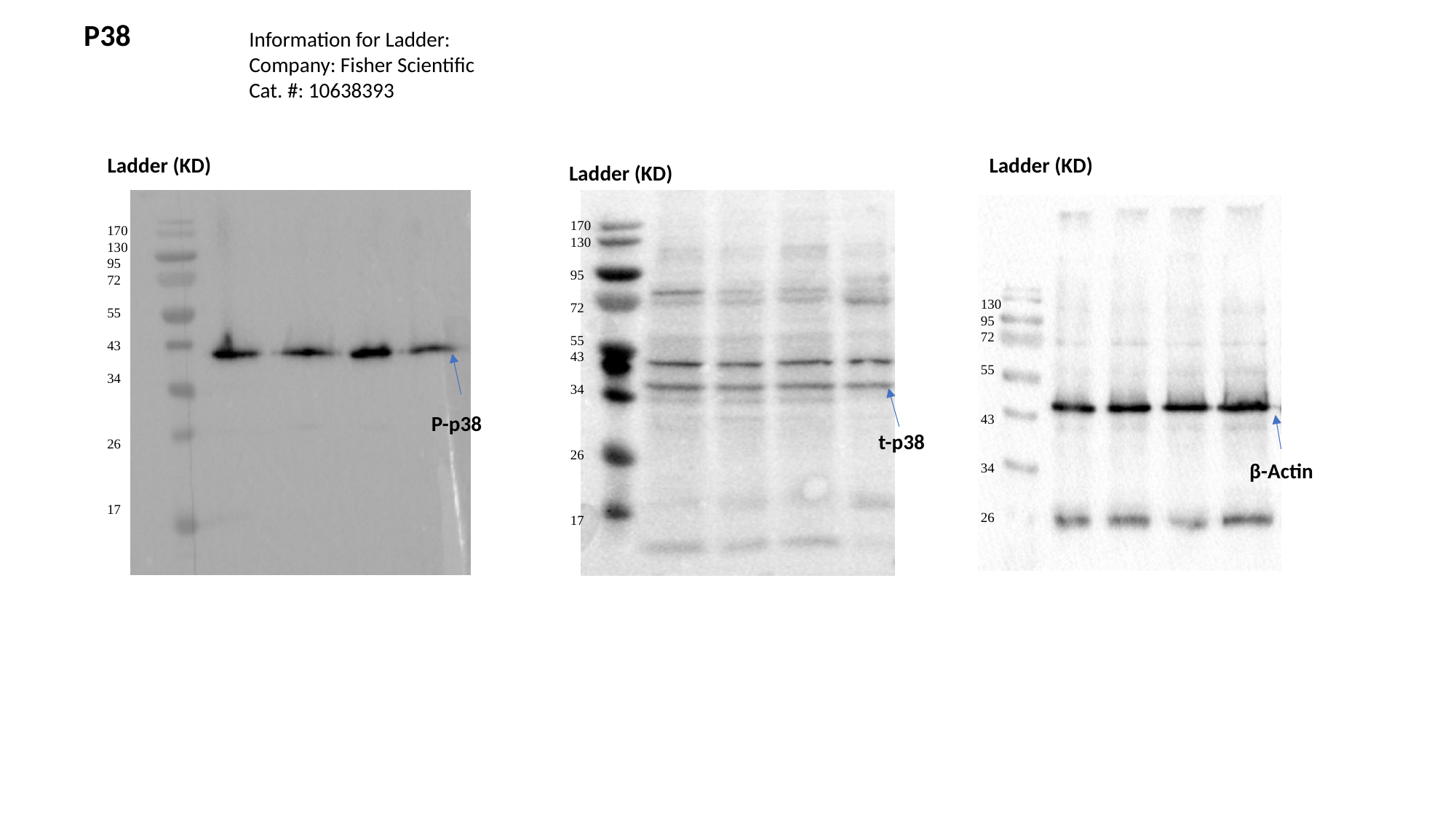

P38
Information for Ladder:
Company: Fisher Scientific
Cat. #: 10638393
Ladder (KD)
Ladder (KD)
Ladder (KD)
170
130
95
72
55
43
34
26
17
170
130
95
72
55
43
34
26
17
130
95
72
55
43
34
26
P-p38
t-p38
β-Actin

## Slide 4
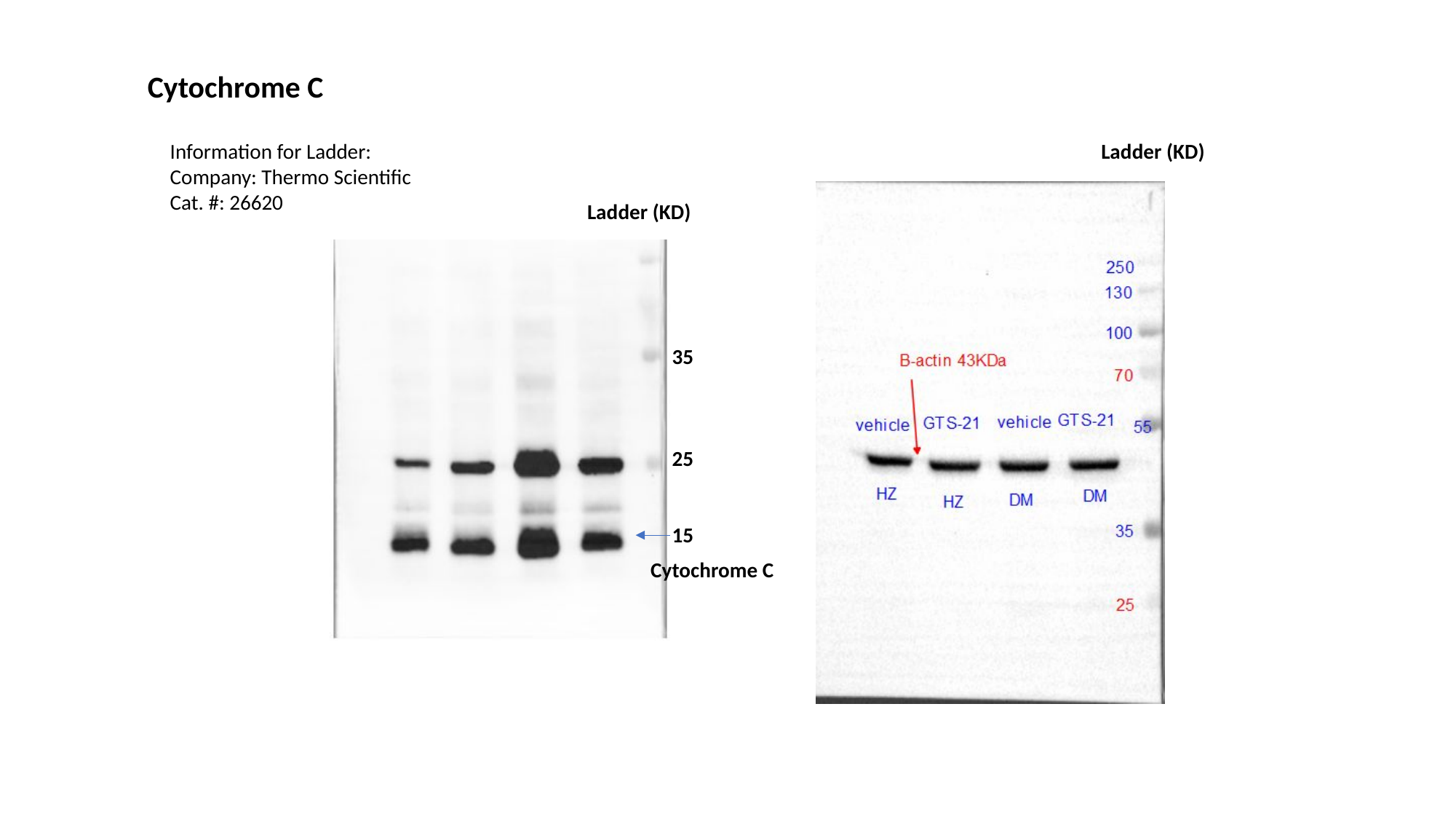

Cytochrome C
Information for Ladder:
Company: Thermo Scientific
Cat. #: 26620
Ladder (KD)
Ladder (KD)
35
25
15
Cytochrome C

## Slide 5
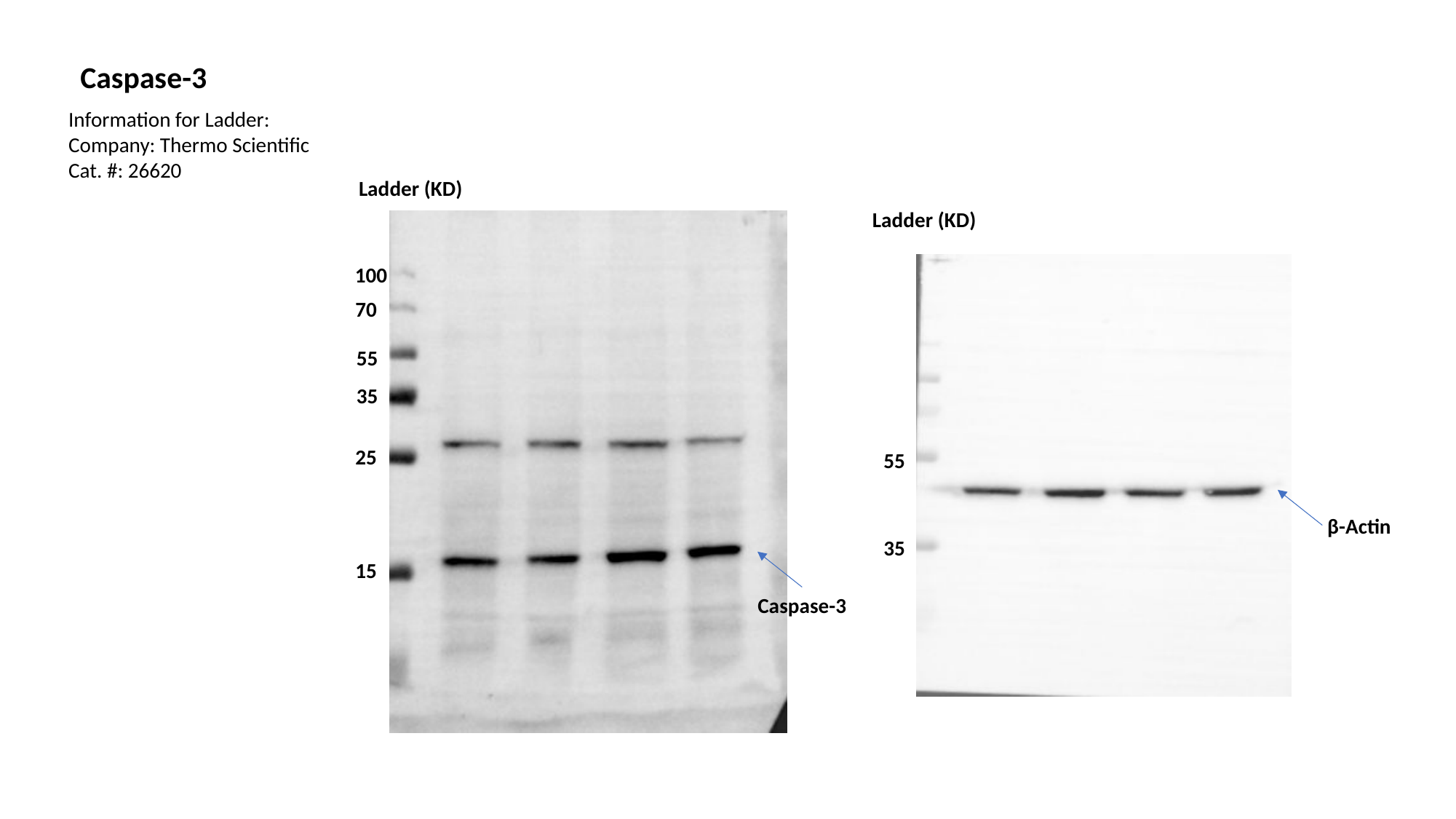

Caspase-3
Information for Ladder:
Company: Thermo Scientific
Cat. #: 26620
Ladder (KD)
Ladder (KD)
100
70
55
35
25
55
β-Actin
35
15
Caspase-3

## Slide 6
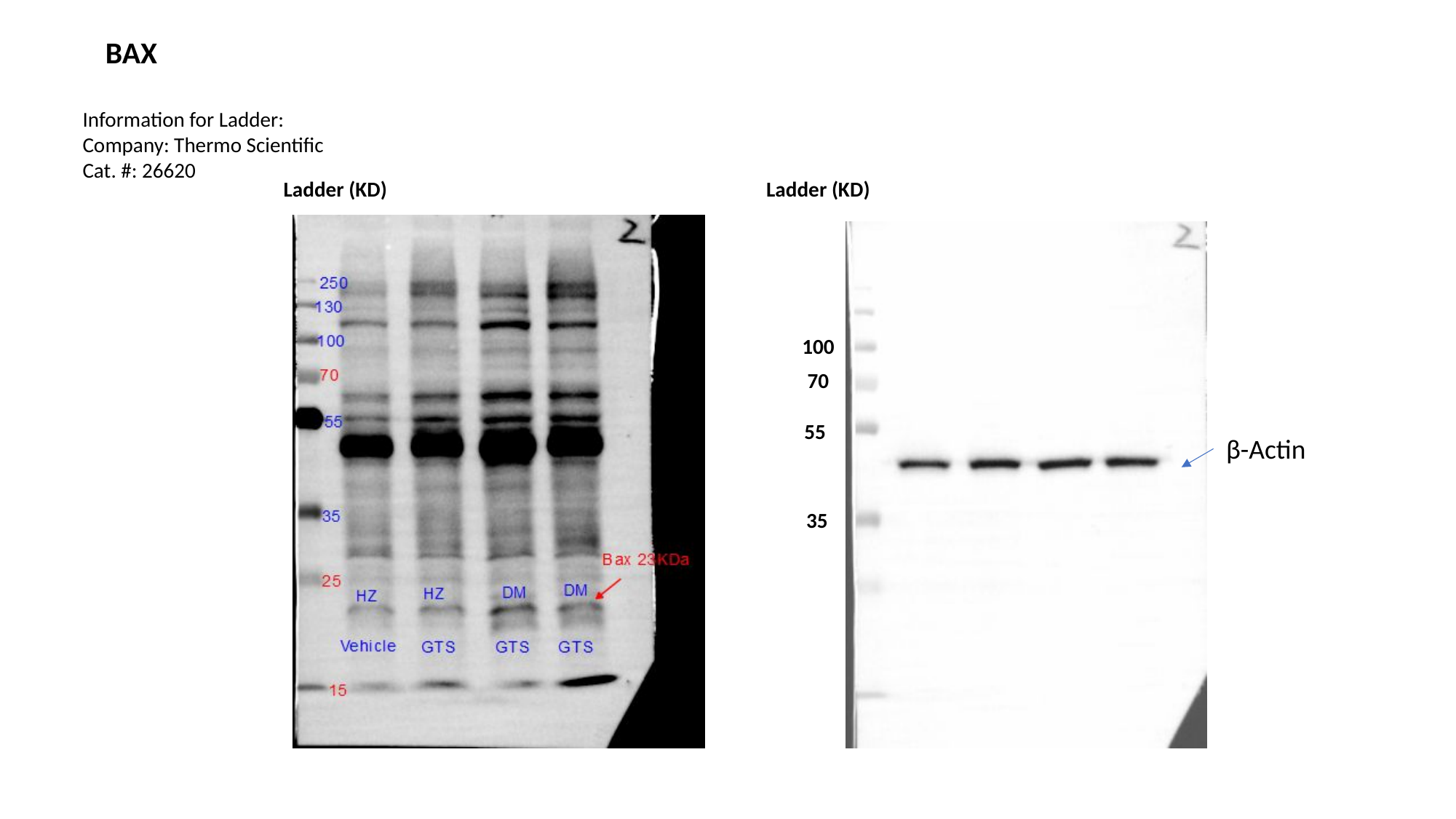

BAX
Information for Ladder:
Company: Thermo Scientific
Cat. #: 26620
Ladder (KD)
Ladder (KD)
100
70
55
β-Actin
35
